# Energy Is Power: Comment on "Energy as a Social and Commercial Determinant of Health: A Qualitative Study of Australian Policy"

**DOI:** 10.34172/ijhpm.8716

**Published:** 2024-12-15

**Authors:** Evelyne de Leeuw

**Affiliations:** Canada Excellence in Research Chair, One Urban Health, Université de Montréal, Montreal, QC, Canada.

**Keywords:** Power, Politics, More-Than-Human, Planet, Balance

## Abstract

Baum et al analyse the Australian energy sector’s influence on health. This commentary suggests that their valuable work may be further enhanced by shifting the gaze in five ways: first, by leaving the health field’s dictate of linear analysis into circularity; second, by removing humans from the centre of analysis; third, by regarding the planet as an indivisible whole system and recognising, for instance, that Australia is not Africa; fourth, by recognising that energy is a source of – sometimes perverse, as demonstrated in the Russia-Ukraine conflict – political power; and fifth, by really starting from the energy point of view rather than the human health perspective. To reorient the debate, the commentary ends with a proposal of the energy cycle as a heuristic to explain global and local health equity.

 The analysis and publication in this journal of a paper by Baum et al^1^ on a healthy energy system are most welcome steps in the exploration of meaningful—commercial and industrial—perspectives on the drivers of human health, and in particular of the health of Australians. Building on their work there are a few possible reflections, comments, and additions.

**First **of all, their analysis – necessarily? – limits itself to a gaze from the public health side of the oculus. The most obvious case in point is the authors’ Figure 3 (*Energy as a Social and Commercial Determinants of Health: Conceptual Framework*). The shape of the programme logic represented in that graph has an uncanny resemblance to frameworks like the health driven PRECEDE-PROCEED framework,^2^ or the World Health Organization (WHO) Conceptual Framework for Action on the Social Determinants of Health.^3^ Such linear stages heuristic is understandable. The health professions and disciplines naturally feel, in linear and causal ways, that “health” is both a key driver as well as outcome of many social, political and ecological processes. As such, they continue to fiercely advocate for health, or perhaps merely the absence of disease. They do so from solid (if not rigid) foundations. But with a shift to a well-being paradigm (which, in fact, is supported by Baum herself)^4^ the linearity of the traditional health thinkers has been challenged. The best case in point is the visual depiction of the well-being economy as a doughnut – a circular paradigm.^5^ So – perhaps human health/well-being should *not* be at the core of an energy discourse^6^ – but rather what energy generation and consumption represent: life.

 The **second** point follows from this. There is growing recognition that an anthropocentric (that is, taking humanity as the core measure of all) view of sustainability is not necessarily the most optimal way to maintain or even improve the world that we live in, together with trillions of other entities and species in a geosphere (the planet’s hardware), atmosphere (airs), hydrosphere (waters) and biosphere (life). This world is continually being compromised by *human*-driven issues.^7^ Energy, as a determinant of life, ought to be a key consideration in planetary and systems ecological concerns, and not limited to human need, as Baum et al posit. The resources for energy in that more comprehensive view are, at their core, not ancient carbon deposits (oil, coal, gas) but indeed higher-level planetary parameters. These include the fact that Terra orbits—together with a bunch of other planets—a star called Sol, that a satellite called Luna orbits Terra, and that this little space system is located somewhere in the suburbs of a galaxy that sits far removed from the centre of the—known or visible/perceived—universe. All of the parameters that (in)directly support life on Terra are created and sustained by that system—sunlight, gravity, mass, and the mysteries of physics that are yet to be revealed to these tiny two-legged spindly creatures from the species Homo that inhabit it, with countless others. We need a bit of a Galileo Galilei 2.0 to take humans out of the centre, and create a more substantive awareness that factors and processes determined by physics (and explained by mathematics) are the true core of planetary existence, balance, energy and life. There is an intricate system of planetary thermodynamics that sustains all—not just humans.^8^

**Third**, although the authors acknowledge an Australian bias, a more global or planetary view would necessarily yield an alternative, and perhaps more politically different, view. For instance, in an analysis of the United Nations Energy Statistics Pocketbook 2022,^9^ a Statista infographic suggests that Africa is leading the world in the deployment of renewables for electricity generation (Figure 1). One could debate the meaningful accuracy of this map. It may well be that renewable consumption per capita is at much higher levels in non-OECD (the Organisation for Economic Co-operation and Development) countries— but we would then fool ourselves in comparing muons with bosons (or apples with oranges). Patterns and levels of energy consumption are as different around the world as are patterns of other consumption. Whether it is water,^10^ space, knowledge or other resources, OECD countries show excessive levels of consumption that are directly related to exceeding planetary boundaries.^11^ Such consumption patterns are depleting the world’s (including the South’s) precious resources.

**Figure 1:**
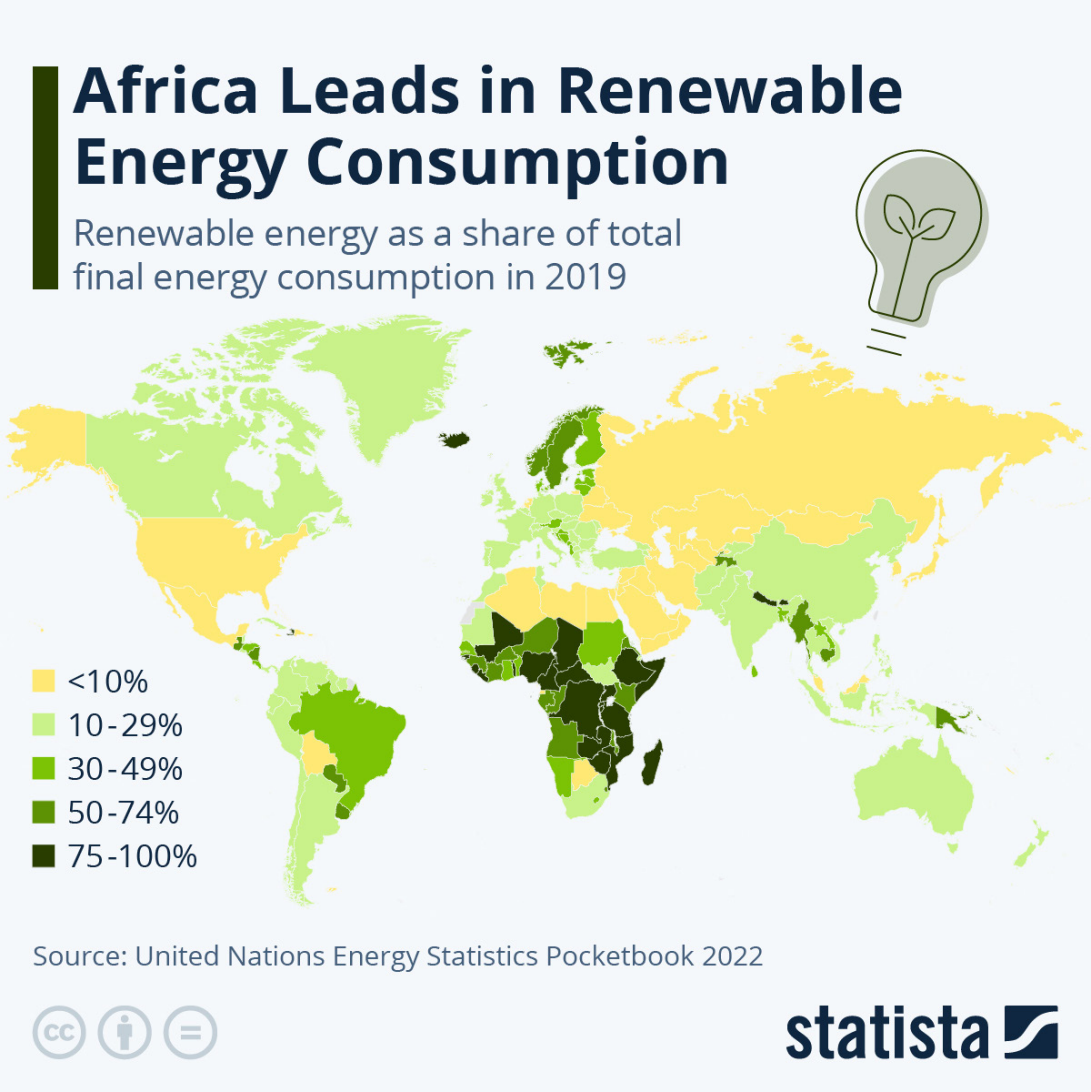
Africa Leads in Renewable Energy Consumption (Statista, 2024, Based on United Nations, 2022).

 Not only do Africans use more total renewable energy – they also use substantially *less* energy per capita than others! This is the key to better understanding not the problems, but the solutions to dilemmas raised by positing energy as a determinant of health (and life): we do not have to be locally better and more equitable, we need to do less – at a planetary scale. And we need to take the Global South for once as an example.

**Fourth**, energy is power. This seems a simple equation, but it is not, especially as the power in my version of the formula is not (just) measured in Joules or Watts. Baum and colleagues, in the energy article and others,^12^ allude to the fact that in Australia (like in many other countries and sub-national areas such as provinces or states) the energy system has been deregulated as a result of the neoliberal mirage. There was a time that much power issues and peripherals (gas and electricity and their storage and distribution parameters, most notably) were considered public utilities. Lighting of the public space, for instance, in many localities is still considered a quintessential public provision – though not always provided with social and health equity considerations in mind.^13^ But most energy production and consumption, since the wave of privatisation and selling off of public assets has taken hold, are no longer considered under the accountable democratic control of the people that are affected by (the accessibility of) energy sources. They have become pawns in the bigger global games of economic domination, infrastructural control, distribution of resources, and the unaccountability, opacity and concentration of capital in the hands of the few. Energy, in a very real and subversive way, is a tool of power. The most perverse confirmation of this perspective is found in the fact that Putin’s Russia in its war with Ukraine primarily targets energy generation and distribution facilities.^14^ Any health equity analysis of energy as a determinant of life on our planet needs to be blatantly specific about who *owns* the power, who *benefits* from it (stock owners or stakeholders?), and what the real financial and opportunity (extraction industries depleting the planetary metabolic and sustainability balance) *costs* are. In health terms, they ought to provide analyses that go beyond the direct proximal health effects of energy (eg, silicosis during mining, chronic obstructive pulmonary disease during consumption of fuels with high concentrations of particulate matter) and address the ill effects on health and well-being of energy poverty and selective accessibility.

 But **fifth **(and I think most important), one could question whether an energy analysis by health scholars published in a health journal would meaningfully shift the policy and industry discourse around the effects of energy generation, supply, and usage on planetary well-being. It is yet again an example of the preaching of an unhappy message to the already-converted parish.^15^ In modern education there is an obsession with “flipping the classroom” (that is, let students determine the learning).^16^ Perhaps it is high time to “flip the communication” – and let the other side of the coin take a lead. There might be many modalities to make that happen, and all transcend a debate around energy as a determinant of health. Examples of flipped communication could include (micro)blogging; using a variety of platforms and channels to put relevant policy briefs in the face of politicians and corporate decision-makers; community mechanisms to hold power (and energy…) to account; partnerships and networking; and redirecting critical intelligence from universities (beholden to the status quo) to knowledge systems that have demonstrated impact, would be a few of these flips.

 Some of the five drawbacks set out above might be resolved by also flipping the gaze of analysis as preliminarily suggested in Figure 2. This depiction of the energy cycle indeed takes it cue from planetary thermodynamics, but recognises that radical pattern shifts are exacted on what used to be a stable system by human-driven energy production, consumption, and waste. Following the important insights provided by the circular economy community,^17^ the ultimate focus should be on total renewables. The “flip” from human-centred to planetary-centred would start to make the argument from the energy side of the equation. For each of the bubbles and connectors in this (globally and locally relevant) energy cycle it should be possible to gauge power distribution consequences for health and equity. The graph also—normatively—suggests that energy *must* be renewable and is not really an infinite resource. In virtually each of the various fields of this energy cycle there is an opportunity to recognise and assess the balance between different modalities, and thus infer the relative advantage of shifting weights on the subsequent parameters, and on health equity. For instance, *energy sourcing, processing*, and* generation* parameters are the result of renewable inputs, extraction industries, and nuclear, hydro, wind and solar generation capacity. As the deliberations at the recent Climate Change Conference of the Parties in Baku have once again showed, considerable power is still vested in extractive industries. Shifting the relative weight of each—either through public choice (consumer driven), policy intervention (government driven) or industry practice—would have consequences for the extraction, storage and distribution patterns required. A poignant current example would be the generation of green hydrogen (that is, the production of fluid hydrogen stores through renewable pathways) and its storage and distribution. This would require vast investments and novel industrial commitments—such as a key long-distance pipeline proposed in the India-Middle East-Europe Economic Corridor.^18^ On the other hand, there is a global development to community ownership of locally generated electricity in distributed governance – shifting, again, power from large corporate actors to citizens and communities.^19^ But energy is not just used by individual and family consumers. Most of it is processed (and perhaps wasted) by the “making industry”—operators that produce anything from telescopes, automobile parts and laboratory equipment to cornflakes, and gravel for tennis courts. These products then need to reach the individual consumer through all imaginable channels the service industry can provide—food delivery, public transport, financial services, etc. In the end, energy expended there enables and limits individual and groups of consumers to make choices for their livelihoods—and how they choose between warmth, cooling, food, drink, transport, housing, etc.

**Figure 2:**
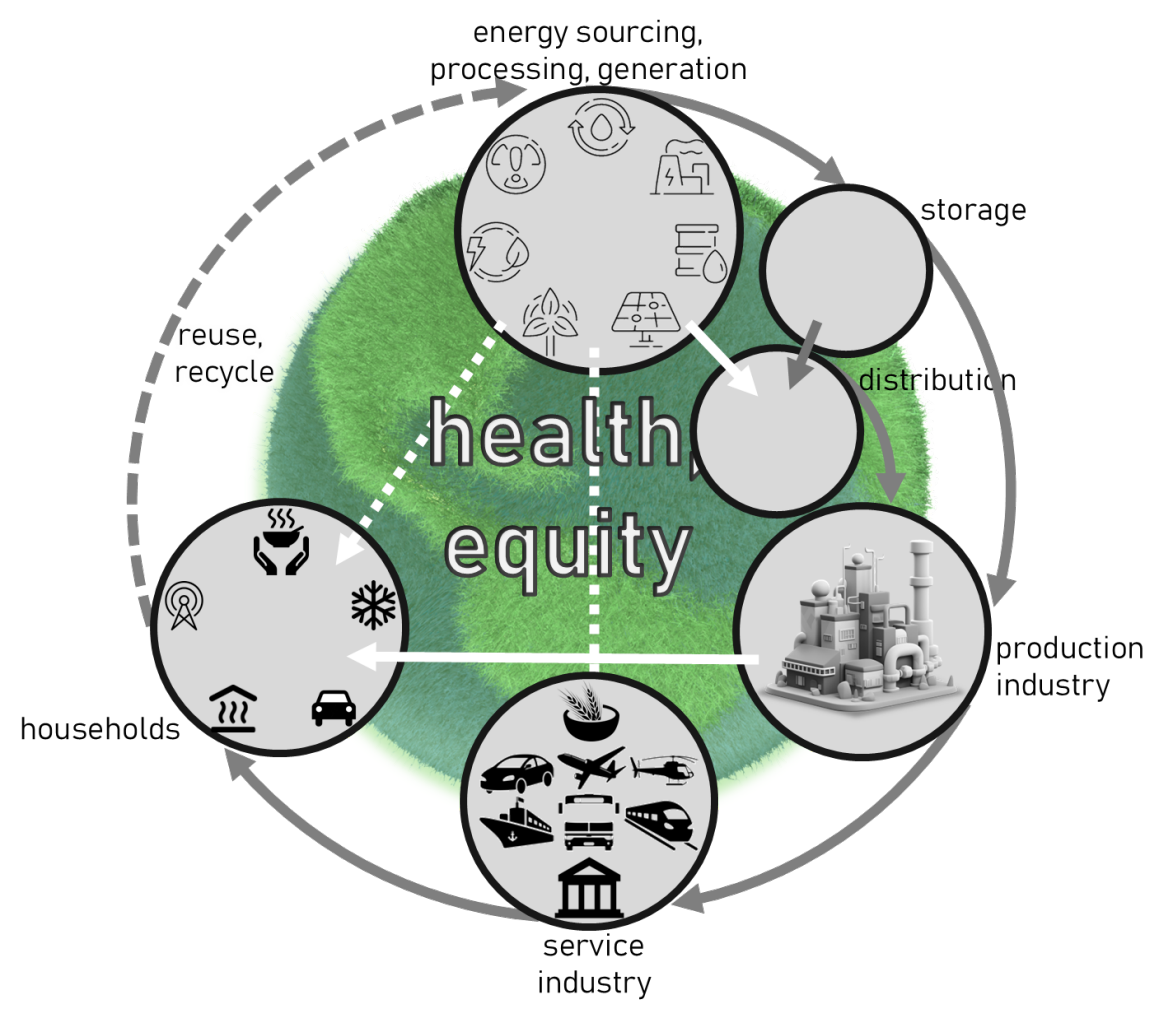
The Energy Cycle, Health Equity, and Planetary Balance.

 Again – each of these separate domains has its own dynamics and balances if not systems homeostasis. But I propose—again—that an analysis from the energy side of the equation rather than the health side of it would allow for a much more powerful discourse and political engagement.

## A Further Call to Action

 In the above I have been profoundly inspired by the original work by Baum and colleagues. I shifted gaze to provide five new perspectives on the energy and health discourse. Ultimately, what my arguments would lead to is the recognition of a need to more meaningfully create and sustain links between an expanded International Energy Agency, the United Nations Environment Programme, the World Bank and WHO. Such linkages are currently absent, and should be informed by empirical work such as the analyses by Baum et al., but enhanced and grown by critical arguments from the global South. As I have demonstrated above, there is much to be learned by OECD/industrialised/high-income countries from efficient energy cycles there.

 Where energy is power, the scholarship in areas that would be considered in the health sector as merely “determinants of health,” such as sustainability, energy, systems ecology, and planetary sciences, would significantly benefit from a well-being (and not just health) perspective. Recent work by Shao^20^ shows how such transdisciplinary and intersectional analyses would have synergy impacts far beyond traditional sectors.

 Third, and related, we have seen, over recent years, a proliferation of the discussion on determinants of health (and well-being) to include political and commercial determinants of health. This is valuable. But as Baum et al have shown, and I have enhanced I hope in the above, the critical ingredient for the transformative success of these discourses are the relational dimensions of the work. In other words: everything depends on everything, and only by recognising the power connections between all elements of this complex well-being system we will fully understand its workings—and opportunities for change.

## Ethical issues

 Not applicable.

## Conflicts of interest

 Author declares that she has no conflicts of interest.
